# Minimal Clinically Important Differences in the Cancer Quality of Life Questionnaires in Patients with Head and Neck Cancer

**DOI:** 10.3390/clinpract14060182

**Published:** 2024-11-01

**Authors:** Athanassios Kyrgidis, Athanasia Printza, Evangelos Vitkos, Konstantinos Lallas, Alexandra Vlassi, Jannis Constantinidis, Stefanos Triaridis

**Affiliations:** 1 Department of Oral & Maxillofacial Surgery, Aristotle University of Thessaloniki Specialized Cancer Treatment and Reconstruction Center, General Hospital of Thessaloniki “George Papanikolaou”, 57010 Thessaloniki, Greece; 2 First University Department of Otolaryngology, Faculty of Medicine, Aristotle University, AHEPA Hospital, 54636 Thessaloniki, Greecetriaridis@hotmail.com (S.T.); 3 Department of Oral and Maxillofacial Surgery, Klinikum Dortmund and Witten/Herdecke University, 44137 Dortmund, Germany; envitkos@gmail.com; 4 Department of Medical Oncology, School of Medicine, Faculty of Health Sciences, Aristotle University, 54124 Thessaloniki, Greece; koplallas@gmail.com; 5 Laboratory of Hygiene, Social & Preventive Medicine and Medical Statistics, Department of Medicine, School of Health Sciences, Aristotle University of Thessaloniki, 54124 Thessaloniki, Greece; alessandra.vlassi@gmail.com

**Keywords:** minimal clinically important difference, oncology, EORTC QLQ-C30, EORTC QLQ-HN35, quality of life

## Abstract

**Purpose**: The purpose of this study is to determine the magnitude of change that is clinically meaningful in the EORTC QLQ-C30 and EORTC QLQ-HN35 instruments in head and neck cancer patients. **Methods**: Two hundred and twenty-two patients completed the EORTC QLQ-C30 and EORTC QLQ-HN35 at baseline and follow-up one to two months later. Minimal clinically important differences (MCIDs) were calculated through anchor- and distribution-based methods for improvement and deterioration. Karnofsky Performance status (KPS) was used as the anchor to determine meaningful change. **Results**: In the group of patients who deteriorated, more scales and symptoms demonstrated statistically significant meaningful change. EORTC QLQ-C30 meaningful change values for deterioration with KPS anchor ranged from 7.2 (physical functioning) to 16.7 units (Global Health Status), and for improvement ranged from 10.0 (role functioning) to 16.7 units (Global Health Status). **Conclusions**: We report—for the first time, to the best of our knowledge—MCID for EORTC QLQ-C30 and QLQ-HN35 in head and neck cancer patients. Knowledge of meaningful change in these questionnaires allows physicians to assess patient change over time, along with evaluating the impact of treatment on quality of life.

## 1. Introduction

Symptom control and health-related quality of life (QoL) are among the most important goals of contemporary oncology practice [1]. The impact of disease and treatment on quality of life is assessed by analysis of the impact of symptoms on patients, which is self-reported by the patients through validated QοL instruments. Mainly, two instruments are used, the EORTC QLQ-C30 and the FACT-G questionnaires. Those QοL assessments include scales of symptoms that influence the majority of patients, along with functional scales, like physical, social, emotional and functional well-being [2,3]. Variation in the reporting of symptoms and pain scores has been reported, signifying that patient QοL self-assessment scores can differentiate with time. Even more, patients have been reported to rate their pain as 3 out of 10 at baseline, and then as 5 out of 10 at follow-up, while at the same time they declared their belief that their pain has improved [4]. Despite the fact that QοL is a central endpoint for oncology patients, the degree of change in these instruments required for the patients to experience deterioration or improvement has not been measured for most types of cancer, including head & neck cancer [1,2,3,5]. Using an adequately statistically powered sample, statistically significant values for these instruments can be recorded, but the clinical significance or otherwise meaningfulness of this change is currently unknown [6]. The aim of this study is to determine the minimal clinically important differences of the EORTC QLQ-C30 and EORTC QLQ-HN35 instruments in a sample of head and neck cancer patients. We utilize both anchor- and distribution-based methods to estimate meaningful changes. Anchor-based methods connect health-related QοL outcomes to established clinical outcomes or to patient perceived rating of improvement or deterioration. It has been reported that anchors must correlate at least moderately (r > 0.30) with health-related QοL, and be interpretable [2,7]. Simpler distribution-based methods rely on the results obtained from the specific study and are interpretations of the central tendency measures (means and standard deviations) of the changes recorded from the study data [8]. Commonly used fractionations include 0.2 standard deviations (SD), 0.3SD, 0.5SD. Most researchers use both anchor and distribution methods in concordance to compare and validate results [8].

## 2. Methods

### 2.1. Patient Population

The study was conducted between 2011 and 2013 in the two academic head & neck departments in Thessaloniki, listed first and second in the affiliation list of the authors. Patients with American Joint Committee on Cancer (AJCC) stage III or IV head and neck cancer who were already enrolled in long-term follow up head and neck oncology settings and who completed the EORTC QLQ-C30 at study baseline and at follow-up (between one to two months later) were eligible. The Karnofsky performance status and patient demographics were recorded at baseline. Baseline for this study was not associated with diagnosis or treatment; rather, the patients were already under follow-up, having had previous surgical and/or radiotherapy and/or chemotherapy modality treatment. The study was approved by the institutional review board and informed consent was obtained from all participants.

### 2.2. Instruments

The EORTC QLQ-C30 is a general QoL questionnaire for cancer patients [9,10,11]. This questionnaire comprises five functional scales (physical functioning, role functioning, emotional functioning, cognitive functioning and social functioning), three symptom scales (fatigue, nausea/vomiting, and pain), and six single items (dyspnea, insomnia, appetite loss, constipation, diarrhea and financial difficulties). All items are scored on a scale from 1 (not at all) to 4 (very much). The questionnaire further includes overall health and overall QoL questions; those are scored on a scale of 1 (very poor) to 7 (excellent). The European Organization for Research and Treatment of Cancer quality of life questionnaire head and neck module (EOTRC QLQ HN35) is a 35-item module. It contains seven symptom scales (pain, swallowing, taste/smell, speech, social eating, social contacts, and sexuality) and six symptom items (teeth problems, trismus, dry mouth, sticky saliva, cough, and feeling ill) [12,13]. These items are all scored on a scale from 1 (not at all) to 4 (very much). Five questions of the EORTC HN35 are answered with a yes or no [14,15]. In both questionnaires, each functional scale, symptom scale, and individual item is converted to a 0–100 scale. A high level of functioning is denoted by a high score on a functional scale. An increase in disease symptoms is denoted by a high score on a symptom scale. The EORTC QLQ-HN35 is considered to be less susceptible to changes in patients’ clinical status when compared to other existing head and neck-specific measures [16]. Both these instruments have formally validated Greek translations [13,16,17].

### 2.3. Statistical Analysis

Anchor- and distribution-based association methods were used in this study, to determine the meaningful change of the EORTC QLQ-C30 with the intent of comparing the two. Demographic descriptive statistics were used. Karnosfky Performance Score was used as anchor, as it has been reported to be an appropriate indicator in this group of patients [18]. Although the instruments have been formerly validated, Cronbach’s alpha and intraclass correlation coefficients (absolute agreement) were used for reliability statistics. Non-parametric Spearman correlation analyses were initially conducted between the KPS anchor and baseline C30 scores to pinpoint symptoms that correlated with moderate or large effect with the KPS anchor. Correlations of at least 0.30 were considered to be moderate, as per Cohen’s rules [19]; then, those items that correlated with moderate or large effect were used in further meaningful clinically important difference analyses [20]. For every one of the symptoms that correlated with the anchor, the mean change in the score was calculated for patients who improved (increased KPS between baseline and Follow-Up—FU), deteriorated (decrease in KPS between baseline and FU) and those who were stable (no change in KPS between baseline and FU). Non-parametric Wilcoxon two-related samples test and 95% confidence intervals (CI) were used to compare values between baseline and follow-up. Estimations of MCID by the distribution method were calculated using 0.2SD, 0.3SD, 0.5SD and 1 Standard Error of Mean (SEM). All calculations were conducted with Statistical Package for Social Sciences (SPSS version 22.0 for Windows).

## 3. Results

We obtained responses from 286 patients who completed the EORTC QLQ-C30 and EORTC QLQ-HN35 questionnaires at baseline. Of them, 222 patients who also completed the questionnaires at a second instance, during follow-up, were eligible for inclusion in the study and are analyzed below. Patient mean age was 68 years (range 36 to 83 years). The majority of patients were male (59.5%), and the most frequent cancer location was the larynx (60.4%) (Table 1). For all questionnaires obtained (baseline and follow-up), Cronbach’s alpha was 0.875 and 0.944 for EORTC QLQ-C30 and EORTC QLQ-HN35, respectively, while intraclass correlation coefficient (absolute agreement) was 0.696 and 0.939 for EORTC QLQ-C30 and EORTC QLQ-HN35, respectively (Table 2). We examined MCID calculations with all functional scales and all symptoms scales. To discriminate between improvement and deterioration, we used a categorical variable of an increase or decrease in KPS. At the last follow-up visit, with regard to their QoL, 63 patients had deteriorated (Table 3), 38 patients had improved (Table 4) and 121 had no change (Table 5).

### 3.1. EORTC QLQ-C30

At baseline, the cognitive functioning scale was rated the least problematic, and the emotional functioning scale was rated the most problematic (Table 2(a)). All functional scales correlated significantly (Spearman’s rho ranged from 0.305 to 0.670) and at least moderately with the KPS anchor; all symptoms correlated significantly (Spearman’s rho ranged from 0.151 to 0.584) with the KPS anchor. The symptoms of fatigue (rho = −0.584), pain (rho = −0.507), insomnia (rho = −0.414), appetite loss (rho = −0.408) and diarrhea (rho = −0.312) also had at least a moderate correlation with the KPS anchor. MCID for deterioration was statistically significant in Global Health/QoL and physical and role functioning scales with unit changes of at least −16.67 (95% CI: −25.58 to −8.75) units on the Global Health/QoL, -7.2 (95% CI: -13.33 to −0.61) on the physical functioning scale, and 9.52 (−17.27 to −1.47) on the role functioning scale (Table 3(a)). The symptoms of nausea/vomiting, pain, constipation and diarrhea were also reported to be significantly aggravated in those patients who deteriorated (Table 3(a)). 

For those 38 patients who improved, changes of 16.67 (95% CI: 6.63 to 26.70) units for Global Health/QoL, 10.09 (95% CI: 1.22 to 18.48) units for role functioning, and 14.25 (95% CI: 1.75 to 26.75) units for emotional functioning were required to constitute statistically significant meaningful improvement (Table 4(a)). No symptoms reached significance for improvement with the present sample. For the 121 patients who remained stable with regard to the KPS anchor, none of the scales or items included in the EORTC QLQ-C30 instrument was found to have statistically significant change (Table 5(a)). The 0.5 SD distribution-based MCID estimates tended to be closer to the MCIDs determined through the KPS anchor (Table 6(a)).

### 3.2. EORTC QLQ-HN35

At baseline, the speech functioning scale was rated the most problematic, and the sexuality and social contact functioning scales were rated the least problematic (Table 2(b)). All HN35 scales correlated significantly and at least moderately with the KPS anchor (Spearman’s rho ranged from 0.30 to 0.45) with the exception of HN speech (rho = −0.12). All symptoms but cough and use of feeding tube correlated significantly (Spearman’s rho ranged from 0.109 to 0.415) with the KPS anchor. The symptoms of HN Teeth (rho = −0.387), mouth opening (rho = −0.354), dry mouth (rho = −0.374), feeling ill (rho = −0.415), painkillers (rho = −0.410) and weight loss (rho 0–0.411) also had at least a moderate correlation with the KPS anchor. 

MCID for deterioration was statistically significant in HN senses 9.79 (95% CI: 0.19 to 19.38) and HN social contact 8.33 (95% CI: 0.61 to 16.04) functional scales (Table 3(b)). The symptom of dry mouth was also reported to be significantly aggravated 12.17 (95% CI: 2.00 to 22.23) in those patients who deteriorated (Table 3(b)). For those 38 patients who improved, no significant changes in HN scales were recorded. The single symptom that demonstrated statistically significant increase was weight gain 34.21 (95% CI: 3.60 to 64.82) (Table 4(b)). For the remaining 121 patients who remained stable with regard to the KPS anchor, none of the scales or items included in the EORTC HN 35 instrument was found to have statistically significant change (Table 5(b)). Contrary to EORTC QLQ-C30, in the EORTC QLQ-HN35 the 0.2 or 0.3 SD distribution-based MCID estimates tended to be closer to the MCIDs determined through the KPS anchor (Table 6(b)).

## 4. Discussion

Determining the meaningful change in QοL assessment is important for practitioners to verify the effectiveness of treatment and whether their patients experience meaningful improvement or deterioration based on their own self-assessment. The KPS anchor-based method was used in the present study along with the distribution-based method. We recorded that MCID on the EORTC QLQ-C30 correlated better to the 0.5SD estimate. The 0.5SD value has been previously reported to reflect most meaningful changes [21]. A 10% decrease in KPS corresponds to an at least 8.75 points decrease in Global Health and Quality of Life (measured with EORTC QLQ-C30), while a 10% increase in KPS corresponds to an at least 6.63 points increase in Global Health and Quality of Life (measured with EORTC QLQ-C30). 

Patients in our study improved or deteriorated in the anticipated direction on the QοL questionnaires. Taken as a whole, in those patients who experienced improvement, the scores on the functional scales increased, while the scores on symptoms scales decreased. The latter described the ability of the symptom and functional scale groups to inversely correlate between them and with patient improvement supports the validity of both instruments. The anchor-based approach for determination of MCID ought to utilize an anchor that is reliable, valid and has an association with the studied QοL tool [22]. KPS encompasses all three properties [18,23] and has demonstrated moderate correlations (r > 0.30) with all the functional scales, and most of the symptom items. KPS also correlates moderately with all but one EORTC QLQ-HN35 scales and most items.

There were relatively few symptoms that showed significant MCID at the one- to two-month follow-up. This could be due to the fact that in a two-month maximum interval between patient self-assessments, disease progress or treatment burden was not adequate to change patients’ opinion with regard to their perceived QoL. Furthermore, head and neck cancer, being a mostly regional disease, may be less suitable to determine MCIDs for QLQ-C30 than other types of cancer with greater metastatic potential, in which more aggressive chemotherapy and hormonotherapy could be administered, i.e. breast cancer. Furthermore, the statistic test used may be too strict or the sample size could render the study underpowered to determine MCID. 

Other studies also included certain palliative oncology patients. Maringwa et al. determined meaningful change in EORTC QLQ-C30 in a group of lung cancer patients [3]. This group also used performance status as the anchor and concluded improvement MCIDs to be 9 for physical functioning, 14 for role functioning, 5 for social functioning, 14 for fatigue and 16 for pain. These values are not very dissimilar to those recorded in the present study, except for pain. The MCID for improvement of pain in the present study is significantly lower than that previously reported by Maringwa. In the present study, we included patients with head and neck cancer who do not often experience severe pain at baseline. Thus, it would not be possible to decrease pain to a larger magnitude [24]. The lung cancer patients that were included in Maringwa’s study have been reported to experience a higher level of pain at baseline, thus they do have the ability to have a larger pain decrease [25]. The same authors also examined the meaningful changes in the EORTC QLQ-C30 in patients with brain cancer. The MCIDs for this group were between 5 and 10, which are on par with the values obtained in this study, despite the fact that the two patient populations experience different symptoms. This difference between meaningful change scores promotes the use of symptom-specific modules such as the EORTC QLQ-HN35 for head and neck cancer. 

Most meaningful change studies use the anchor- based method, but few elaborate on the suitability of the anchor used. It has been supported that the use of the anchor-based method must depend upon the association that the anchor has with the studied QoL instrument [22] and that empirical validation of each anchor ought to be examined before its use. In most meaningful change studies, the correlation between the anchor and the QoL score is calculated through the Spearman or Pearson correlation coefficient. Some researchers have used linear regression to plot changes in QoL scores as a function of anchor changes [8]. Limitations pertain to both approaches. Using the correlation coefficient appears more meaningful when the anchor is of a continuous nature, like KPS in the present study, and its use when the anchor is categorical is questionable. The linear regression approach could be restrictive if the relationship between the anchor and QoL is non-linear, [26,27] a very plausible scenario in this study. Thus, the suitability of specific anchors for the determination of MCIDs remains a field for further study. Some investigators [20] have recommended identifying minimally important change on the anchor and restricting the analyses to this subgroup of patients [3]. Thus, it can be suggested that the approach used in the current paper evaluates a threshold for minimal change, defined by the numerical change in KPS. Relying on KPS status as the anchor variable might be problematic; however, due to the lack of information about other relevant anchors [18,23] (including some that might correspond more closely to QOL changes), it appears to be the most generalizable choice.

Limitations to this study are the inclusion of head and neck cancer patients with a variety of primary tumor locations. On the other hand, previous studies concluded that inclusion of more dissimilar patient populations when determining the meaningful changes of the EORTC QLQ-30 could potentially prevent some symptom scales from reaching statistically significant correlation with the anchor. Another common limitation to clinically meaningful change studies is “response shift”: patients, when informed of their diagnosis, might change their personal frame of reference with regard to QoL [28]. Furthermore, the low number of patients who experienced improvement or deterioration could potentially affect the values obtained for MCID. 

## 5. Conclusions

Awareness of the minimal amount of change in the QoL instrument that is necessary for the patients to experience clinically relevant improvement or deterioration is important when assessing clinical trial outcomes. Furthermore, awareness of the anticipated meaningful change can assist researchers in determining the sample size required for clinical trials. More studies in other populations, employing the use of common anchors such as performance status, are needed to better determine MCIDs. These studies might also divide up the patient population and group patients who experience similar symptoms together. Other EORTC symptom-specific modules should also be employed in these patient populations and undergo meaningful change studies, as symptom-specific modules may prove to be more useful in these specific patient populations than the generic EORTC QLQ-C30. 

## Figures and Tables

**Table 1:** Patient demographics.

	Baseline	FU
n	222	
Age Mean ± SD	65.5 ± 9.4	
Age Median (range)	68.0 (36–83)	
**Location of Tumor**		
Oral	43	
Rhinopharynx	18	
Oropharynx	10	
Larynx	134	
Neck	6	
Parotid	8	
Nasal-Sinus	3	
**Treatment Modality**		
Surgery alone	34	
Radiotherapy alone	34	
Chemotherapy alone	4	
Surgery with radiotherapy	46	
Surgery with radiochemotherapy	56	
Radiochemotherapy without surgery	48	
**KPS**		
Mean ± SD	63.38 ± 9.12	62.25 ± 9.14
**Gender**		
Female	90 (40.5%)	
Male	132 (59.5%)	

**Table 2:** (**a**) EORTC QLQ-C30 scores at baseline and at follow-up. (**b**) EORTC QLQ-HN35 scores at baseline and at follow-up.

(a)						
TimePoint	Baseline		FU		Mean Change [FU-Baseline]	*p*
	Mean	Std. Deviation	Mean	Std. Deviation		
Global health status/QoL	60.85	15.34	59.83	18.93	−1.01	0.690
Physical Function	83.96	14.02	82.91	13.56	−1.05	0.277
Role Function	89.19	16.58	88.21	15.93	−0.98	0.312
Emotional Function	74.10	21.25	75.34	20.26	1.24	0.506
Cognitive Function	91.44	13.80	90.77	13.49	−0.68	0.417
Social Function	85.76	17.13	85.37	16.69	−0.39	0.669
Fatigue	17.42	17.08	18.07	16.71	0.65	0.592
Nausea/vomiting	5.41	10.31	9.61	12.89	4.20	0.000
Pain	10.36	17.52	12.54	17.35	2.18	0.049
Dyspnoea	10.81	19.11	11.41	18.76	0.60	0.592
Insomnia	15.32	25.28	15.47	24.28	0.15	0.733
Appetite loss	9.91	17.13	11.86	17.78	1.95	0.193
Constipation	4.50	11.42	9.61	15.13	5.11	0.000
Diarrhoea	7.21	17.60	11.11	18.94	3.90	0.004
Financial problems	16.68	18.36	16.99	18.36	0.30	0.853
**(b)**						
**TimePoint**	**Baseline**		**FU**			
	**Mean**	**Std. Deviation**	**Mean**	**Std. Deviation**	**Mean Change [FU-Baseline]**	***p***
HN Pain	18.02	17.85	17.12	17.67	−0.90	0.510
HN Swallowing	18.92	23.91	17.42	22.30	−1.50	0.797
HN Senses	17.12	20.36	18.99	19.63	1.88	0.189
HN Speech	29.43	35.33	29.38	31.91	−0.05	0.388
HN Social eating	18.47	19.91	19.96	19.71	1.49	0.293
HN Social contact	14.11	17.69	16.22	17.38	2.11	0.046
HN Sexuality	12.79	12.33	13.32	13.19	0.53	0.879
HN Teeth	15.32	22.77	16.74	22.71	1.43	0.405
HN Opening mouth	11.71	22.26	12.19	21.34	0.48	0.542
HN Dry mouth	15.32	19.95	19.00	21.57	3.69	0.071
HN Sticky saliva	16.22	24.09	18.98	25.41	2.77	0.243
HN Coughed	22.52	30.14	24.92	30.45	2.40	0.343
HN Felt ill	17.12	24.09	17.73	23.92	0.61	0.714
HN Painkillers	35.14	47.85	35.00	47.81	−0.14	0.976
HN Nutritional supp.	13.51	34.26	13.96	34.74	0.45	0.890
HN Feeding tube	8.11	27.36	9.01	28.70	0.90	0.735
HN Weight loss	27.03	44.51	29.73	45.81	2.70	0.528
HN Weight gain	21.62	41.26	27.48	44.74	5.86	0.152

**Table 3:** (**a**) EORTC QLQ-C30. (**b**) EORTC QLQ-HN35. MCIDs for KPS anchor. Patients who deteriorated (n = 63).

(a)												
TimePoint	Baseline				FU				Deterioration			
	Mean	Std. Deviation	Low 95% CI	High 95% CI	Mean	Std. Deviation	Low 95% CI	High 95% CI	Mean Change [FU-Baseline]	Low 95% CI	High 95% CI	*p*
Global health status/QoL	59.79	15.74	55.83	63.75	43.12	15.74	39.17	47.08	−16.67	**−24.58**	**−8.75**	0.000
Physical Function	83.92	14.25	80.33	87.05	76.72	11.91	73.72	79.72	−7.20	**−13.33**	**−0.61**	0.001
Role Function	90.21	15.45	86.32	94.10	80.69	15.32	76.83	84.85	−9.52	**−17.27**	**−1.47**	0.000
Emotional Function	73.81	20.35	68.68	78.93	69.58	17.08	65.28	73.88	−4.23	−13.65	5.20	0.326
Cognitive Function	92.06	13.34	88.70	95.42	87.04	14.18	83.47	90.61	−5.03	−11.95	1.91	0.050
Social Function	86.81	16.16	82.74	90.88	81.75	16.04	77.71	85.79	−5.07	−13.17	3.05	0.050
Fatigue	17.46	17.71	13.00	21.92	24.16	15.91	20.16	28.17	6.70	−1.76	15.17	0.050
Nausea/vomiting	4.50	10.02	1.97	7.02	20.63	12.60	17.46	23.81	16.14	**10.44**	**21.84**	0.000
Pain	10.05	17.08	5.75	14.35	19.58	16.27	15.48	23.67	9.52	**1.13**	**17.92**	0.000
Dyspnea	9.52	18.38	4.89	14.15	16.40	19.74	11.43	21.37	6.88	−2.72	16.48	0.050
Insomnia	15.87	26.00	9.33	22.42	21.69	25.51	15.27	28.12	5.82	−7.15	18.79	0.088
Appetite loss	8.47	15.80	4.49	12.45	16.40	17.83	11.91	20.89	7.94	−0.54	16.40	0.050
Constipation	3.70	10.56	1.04	6.36	21.69	16.02	17.66	25.73	17.99	**11.30**	**24.69**	0.000
Diarrhea	5.82	15.31	1.96	9.68	21.16	18.26	16.57	25.76	15.34	**6.89**	**23.80**	0.000
Financial problems	16.46	17.78	11.98	20.94	18.05	17.73	13.58	22.51	1.59	−7.36	10.53	0.600
**(b)**												
**TimePoint**	**Baseline**				**FU**							
	**Mean**	**Std. Deviation**	**Low 95% CI**	**High 95% CI**	**Mean**	**Std. Deviation**	**Low 95% CI**	**High 95% CI**	**Mean Change [FU-Baseline]**	**Low 95% CI**	**High 95% CI**	***p***
HN Pain	19.18	18.55	14.51	23.85	19.31	18.56	14.64	23.99	0.13	−9.21	9.48	0.956
HN Swallowing	16.27	22.92	10.50	22.04	17.46	22.49	11.80	23.12	1.19	−10.24	12.62	0.537
HN Senses	14.81	19.19	9.98	19.65	24.60	18.90	19.84	29.36	9.79	**0.19**	**19.38**	**0.002**
HN Speech	23.10	32.50	14.92	31.29	31.39	26.81	24.64	38.15	8.29	−6.65	23.23	0.089
HN Social eating	17.33	18.59	12.65	22.01	25.57	16.91	21.32	29.83	8.25	−0.69	17.18	0.066
HN Social contact	11.70	15.81	7.72	15.68	20.02	14.84	16.29	23.76	8.33	**0.61**	**16.04**	**0.000**
HN Sexuality	13.28	12.69	10.08	16.47	16.09	13.73	12.63	19.55	2.82	−3.84	9.47	0.310
HN Teeth	16.40	23.85	10.40	22.41	25.93	25.00	19.63	32.22	9.52	−2.78	21.82	0.061
HN Opening mouth	11.11	21.59	5.67	16.55	17.46	22.29	11.85	23.07	6.35	−4.70	17.40	0.057
HN Dry mouth	12.70	18.38	8.07	17.33	24.87	21.56	19.33	30.30	12.17	**2.00**	**22.23**	**0.001**
HN Sticky saliva	12.70	21.94	7.12	18.22	23.28	25.84	16.77	29.79	10.58	−1.45	22.67	0.057
HN Coughed	17.99	27.32	11.11	28.87	29.10	29.02	21.79	36.41	11.11	−7.08	25.30	0.056
HN Felt ill	15.87	23.08	10.06	21.68	23.28	25.84	16.77	29.79	7.41	−4.91	19.73	0.082
HN Painkillers	38.10	48.95	25.77	50.42	41.27	49.63	28.77	53.77	3.17	−21.65	28.00	0.717
HN Nutritional supp.	14.29	35.27	5.40	23.17	17.46	38.27	7.82	27.10	3.17	−15.35	21.70	0.627
HN Feeding tube	6.35	24.58	0.16	12.54	6.35	24.58	0.16	12.54	0.00	−12.38	12.38	1.000
HN Weight loss	26.98	44.74	15.72	38.25	46.03	50.24	33.38	58.69	19.05	−4.87	42.97	0.070
HN Weight gain	20.63	40.79	10.36	30.91	19.05	39.58	9.08	29.02	−1.59	−21.83	18.66	0.824

Statistical significance typed in bold.

**Table 4:** (**a**) EORTC QLQ-C30. (**b**) EORTC QLQ-HN35. MCIDs for KPS anchor. Patients who improved (n = 38).

(a)												
TimePoint	Baseline				FU				Improvement			
	Mean	Std. Deviation	Low 95% CI	High 95% CI	Mean	Std. Deviation	Low 95% CI	High 95% CI	Mean Change [FU-Baseline]	Low 95% CI	High 95% CI	*p*
Global health status/QoL	60.96	15.27	55.95	65.98	77.63	15.27	72.61	82.65	16.67	**6.63**	**26.70**	0.000
Physical Function	83.16	14.85	78.28	88.04	88.95	11.83	85.06	92.84	5.79	−2.98	14.56	0.080
Role Function	87.72	18.86	81.52	93.92	97.81	6.90	95.54	100.00	10.09	**1.62**	**18.48**	0.004
Emotional Function	73.03	22.21	65.73	80.33	87.28	15.83	82.08	92.48	14.25	**1.75**	**26.75**	0.004
Cognitive Function	90.35	15.32	85.32	95.39	94.74	10.33	91.34	98.13	4.39	−4.05	12.81	0.236
Social Function	84.21	18.15	78.24	90.18	90.35	14.31	85.65	95.05	6.14	−4.53	16.81	0.140
Fatigue	18.13	17.60	12.34	23.91	10.82	14.72	5.98	15.66	−7.31	−17.93	3.32	0.050
Nausea/vomiting	5.70	10.45	2.27	9.14	3.51	8.80	0.62	6.40	−2.19	−8.52	4.13	0.273
Pain	10.96	17.87	5.09	16.84	7.89	14.36	3.17	12.62	−3.07	−13.67	7.53	0.527
Dyspnea	12.28	21.11	5.34	19.22	4.39	13.80	0.00	8.92	−7.89	−19.22	3.58	0.050
Insomnia	14.91	25.35	6.58	23.24	6.14	15.22	1.14	11.14	−8.77	−22.10	4.56	0.092
Appetite loss	10.53	17.51	4.77	16.28	8.77	16.77	3.26	14.29	−1.75	−13.02	9.52	0.616
Constipation	4.39	11.42	0.63	8.14	4.39	11.42	0.63	8.14	0.00	−7.51	7.51	1.000
Diarrhea	8.77	20.04	2.19	15.36	6.14	17.08	0.53	11.75	−2.63	−14.83	9.56	0.528
Financial problems	18.13	19.91	11.59	24.68	17.25	19.96	10.69	23.82	−0.88	−13.99	12.23	0.830
**(b)**												
**TimePoint**	**Baseline**				**FU**							
	**Mean**	**Std. Deviation**	**Low 95% CI**	**High 95% CI**	**Mean**	**Std. Deviation**	**Low 95% CI**	**High 95% CI**	**Mean Change [FU-Baseline]**	**Low 95% CI**	**High 95% CI**	***p***
HN Pain	17.54	18.05	11.61	23.48	11.84	16.28	6.49	17.19	−5.70	−16.99	5.580	0.073
HN Swallowing	19.30	23.97	11.42	27.18	9.21	11.91	5.30	13.13	−10.09	−21.88	1.710	0.151
HN Senses	18.42	21.85	11.24	25.60	12.72	16.64	7.25	18.19	−5.70	−18.35	6.950	0.335
HN Speech	30.99	36.55	18.98	43.01	18.42	26.25	9.79	27.05	−12.57	−33.22	8.070	0.158
HN Social eating	19.30	20.51	12.56	26.04	13.60	18.42	7.54	19.65	−5.70	−18.50	7.090	0.262
HN Social contact	14.30	17.63	8.51	20.10	10.62	14.47	5.86	15.37	−3.68	−14.24	6.860	0.532
HN Sexuality	12.47	11.74	8.61	16.33	9.45	10.26	6.07	12.82	−3.02	−10.26	4.210	0.286
HN Teeth	14.91	22.86	7.40	22.42	10.53	20.66	3.74	17.32	−4.39	−18.68	9.920	0.331
HN Opening mouth	11.40	22.30	4.08	18.73	6.14	17.08	0.53	11.75	−5.26	−18.20	7.670	0.236
HN Dry mouth	14.91	20.06	8.32	21.50	13.16	19.82	6.64	19.67	−1.75	−14.86	11.350	0.659
HN Sticky saliva	15.79	24.18	7.84	23.74	13.16	22.65	7.71	20.60	−2.63	−16.03	12.760	0.616
HN Coughed	22.81	30.12	12.91	32.71	16.67	29.76	6.88	26.45	−6.14	−25.83	13.540	0.224
HN Felt ill	17.54	24.18	9.60	25.49	8.77	14.88	3.88	13.66	−8.77	−21.61	4.060	0.106
HN Painkillers	34.21	48.08	18.41	50.01	21.05	41.32	7.47	34.63	−13.16	−42.54	16.220	0.203
HN Nutritional supp.	13.16	34.26	1.90	24.42	10.53	31.10	0.30	20.75	−2.63	−24.12	18.850	0.724
HN Feeding tube	10.53	31.10	0.30	20.75	10.53	31.10	0.30	20.75	0.00	−20.45	20.450	1.000
HN Weight loss	26.32	44.63	11.65	40.98	13.16	34.26	1.90	24.42	−13.16	−39.08	12.770	0.152
HN Weight gain	23.68	43.09	9.52	37.85	57.89	50.04	41.45	74.34	34.21	**3.60**	**64.820**	**0.003**

Statistical significance typed in bold.

**Table 5:** (**a**) EORTC QLQ-C30. (**b**) EORTC QLQ-HN35. MCIDs for KPS anchor. Patients who remained stable (n = 121).

(a)												
TimePoint	Baseline				FU				No Change			
	Mean	Std. Deviation	Low 95% CI	High 95% CI	Mean	Std. Deviation	Low 95% CI	High 95% CI	Mean Change [FU-Baseline]	Low 95% CI	High 95% CI	*p*
Global health status/QoL	61.36	15.25	58.62	64.11	62.95	14.27	60.38	65.52	1.58	−3.73	6.90	0.381
Physical Function	84.24	13.74	81.77	86.72	84.30	13.63	81.84	86.75	0.06	−4.88	4.98	0.993
Role Function	89.12	16.49	86.15	92.09	89.26	15.79	86.41	92.10	0.14	−5.68	5.95	0.948
Emotional Function	74.59	21.57	70.70	78.47	75.28	21.41	71.42	79.13	0.69	−7.05	8.43	0.783
Cognitive Function	91.46	13.63	89.01	93.91	90.77	14.10	88.23	93.31	−0.69	−5.68	4.30	0.693
Social Function	85.69	17.39	82.56	88.82	85.28	17.50	82.13	88.43	−0.41	−6.69	5.87	0.857
Fatigue	17.17	16.73	14.16	20.18	16.53	16.67	13.53	19.53	−0.64	−6.65	5.37	0.742
Nausea/vomiting	5.79	10.48	3.90	7.67	6.06	10.76	4.12	8.00	0.28	−3.55	4.10	0.866
Pain	10.33	17.78	7.13	13.53	10.74	17.85	7.53	13.96	0.41	−6.00	6.83	0.803
Dyspnea	11.02	18.95	7.61	14.43	10.19	18.68	6.83	13.56	−0.83	−7.60	5.95	0.682
Insomnia	15.15	25.09	10.64	19.67	15.43	25.11	10.91	19.95	0.28	−8.76	9.31	0.905
Appetite loss	10.47	17.76	7.27	13.67	11.02	17.94	7.79	14.25	0.55	−5.88	6.98	0.786
Constipation	4.96	11.91	2.81	7.10	4.41	11.34	2.37	6.45	−0.55	−4.73	3.64	0.712
Diarrhea	7.44	18.00	4.20	10.68	7.71	18.64	4.36	11.07	0.28	−6.32	6.87	0.977
Financial problems	16.34	18.29	12.92	19.50	16.34	18.29	12.92	19.50	0.00	−6.58	6.58	1.000
(**b**)												
**TimePoint**	**Baseline**				**FU**							
	**Mean**	**Std. Deviation**	**Low 95% CI**	**High 95% CI**	**Mean**	**Std. Deviation**	**Low 95% CI**	**High 95% CI**	**Mean Change [FU-Baseline]**	**Low 95% CI**	**High 95% CI**	***p***
HN Pain	17.56	17.54	13.70	20.42	17.63	17.42	13.21	21.84	0.07	−7.21	8.14	0.939
HN Swallowing	20.18	24.48	15.09	24.38	19.97	24.14	13.04	24.70	−0.21	−11.34	9.61	0.986
HN Senses	17.91	20.54	13.97	21.88	15.69	20.25	10.86	20.51	−2.22	−11.02	6.54	0.916
HN Speech	32.23	36.22	24.38	38.09	29.58	35.37	21.51	37.65	−2.65	−16.58	13.27	0.984
HN Social eating	18.80	20.51	14.52	22.42	17.93	20.80	12.86	23.01	−0.87	−9.56	8.49	0.945
HN Social contact	15.30	18.61	10.95	17.95	14.98	18.99	10.52	19.44	−0.32	−7.43	8.49	0.742
HN Sexuality	12.62	12.43	10.22	15.01	13.00	13.61	9.59	16.21	0.38	−5.42	5.99	0.953
HN Teeth	14.88	22.34	10.83	19.36	15.20	20.65	10.09	20.31	0.32	−9.27	9.48	0.842
HN Opening mouth	12.12	22.77	7.81	16.72	11.30	21.58	5.95	16.60	−0.82	−10.77	8.79	0.875
HN Dry mouth	16.80	20.69	12.90	21.06	15.20	20.65	10.09	20.31	−1.60	−10.97	7.41	0.776
HN Sticky saliva	18.18	25.09	13.69	23.41	17.65	25.82	11.66	23.64	−0.53	−11.75	9.95	0.980
HN Coughed	24.79	31.48	18.64	31.05	25.36	31.15	18.52	34.42	0.56	−12.53	15.78	0.838
HN Felt ill	17.63	24.75	12.27	21.69	17.65	24.48	10.67	22.66	0.02	−11.02	10.39	0.954
HN Painkillers	33.88	47.53	24.80	43.13	33.82	48.24	22.29	45.36	−0.06	−20.84	20.56	0.715
HN Nutritional supp.	13.22	34.02	5.92	18.61	13.22	34.02	3.91	19.62	0.00	−14.70	13.70	1.000
HN Feeding tube	8.26	27.65	1.80	11.41	9.92	30.01	1.91	15.74	1.65	−9.50	13.94	0.655
HN Weight loss	27.27	44.72	19.59	37.02	29.41	44.29	18.30	40.52	2.14	−18.72	20.93	0.885
HN Weight gain	19.81	41.24	12.10	27.52	17.65	38.40	8.35	26.94	−2.16	−19.17	14.84	0.877

**Table 6:** (**a**) EORTC QLQ-C30. (**b**) EORTC QLQ-HN35. Distribution-based MCID.

(a)								
EORTC QLQ-C30		Baseline				FU		
	0.2 SD	0.3 SD	0.5 SD	SEM	0.2 SD	0.3 SD	0.5 SD	SEM
Global health status/QoL	3.07	4.60	7.67	1.03	3.79	5.68	9.47	1.27
Physical Function	2.80	4.21	7.01	0.94	2.71	4.07	6.78	0.91
Role Function	3.32	4.97	8.29	1.11	3.19	4.78	7.97	1.04
Emotional Function	4.25	6.38	10.63	1.43	4.05	6.08	10.13	1.35
Cognitive Function	2.76	4.14	6.90	0.93	2.70	4.05	6.75	0.92
Social Function	3.43	5.14	8.56	1.15	3.34	5.01	8.35	1.12
Fatigue	3.42	5.13	8.54	1.15	3.34	5.01	8.36	1.12
Nausea / vomiting	2.06	3.09	5.16	0.69	2.58	3.87	6.45	0.87
Pain	3.50	5.26	8.76	1.18	3.47	5.20	8.67	1.17
Dyspnea	3.82	5.73	9.56	1.28	3.75	5.63	9.38	1.25
Insomnia	5.06	7.58	12.64	1.70	4.86	7.28	12.14	1.63
Appetite loss	3.43	5.14	8.57	1.15	3.56	5.33	8.89	1.20
Constipation	2.28	3.43	5.71	0.77	3.03	4.54	7.57	1.01
Diarrhea	3.52	5.28	8.80	1.18	3.79	5.68	9.47	1.29
Financial problems	3.67	5.51	9.18	1.23	3.67	5.51	9.18	1.23
(**b**)								
**EORTC QLQ-HN35**		**Baseline**				**FU**		
	**0.2 SD**	**0.3 SD**	**0.5 SD**	**SEM**	**0.2 SD**	**0.3 SD**	**0.5 SD**	**SEM**
HN Pain	3.57	5.36	8.93	1.20	3.53	5.30	8.83	1.19
HN Swallowing	4.78	7.17	11.96	1.60	4.46	6.69	11.15	1.50
HN Senses	4.07	6.11	10.18	1.37	3.93	5.89	9.82	1.32
HN Speech	7.07	10.60	17.67	2.37	6.38	9.57	15.95	2.16
HN Social eating	3.98	5.97	9.96	1.34	3.94	5.91	9.85	1.32
HN Social contact	3.54	5.31	8.84	1.19	3.48	5.22	8.69	1.17
HN Sexuality	2.47	3.70	6.17	0.86	2.64	3.96	6.60	0.97
HN Teeth	4.55	6.83	11.39	1.53	4.54	6.81	11.35	1.54
HN Opening mouth	4.45	6.68	11.13	1.49	4.27	6.40	10.67	1.45
HN Dry mouth	3.99	5.98	9.97	1.34	4.31	6.47	10.79	1.45
HN Sticky saliva	4.82	7.23	12.05	1.62	5.08	7.62	12.71	1.73
HN Coughed	6.03	9.04	15.07	2.02	6.09	9.14	15.23	2.06
HN Felt ill	4.82	7.23	12.05	1.62	4.78	7.18	11.96	1.61
HN Painkillers	9.57	14.35	23.92	3.21	9.56	14.34	23.90	3.22
HN Nutritional supp.	6.85	10.28	17.13	2.30	6.95	10.42	17.37	2.33
HN Feeding tube	5.47	8.21	13.68	1.84	5.74	8.61	14.35	1.93
HN Weight loss	8.90	13.35	22.26	2.99	9.16	13.74	22.91	3.07
HN Weight gain	8.25	12.38	20.63	2.77	8.95	13.42	22.37	3.00

## Data Availability

More data and results are available at the Aristotle University Repository, https://ikee.lib.auth.gr/record/131971/ [accessed date 18 September 2024].
